# Association between Common Germline Genetic Variation in 94 Candidate Genes or Regions and Risks of Invasive Epithelial Ovarian Cancer

**DOI:** 10.1371/journal.pone.0005983

**Published:** 2009-06-19

**Authors:** Lydia Quaye, Jonathan Tyrer, Susan J. Ramus, Honglin Song, Eva Wozniak, Richard A. DiCioccio, Valerie McGuire, Estrid Høgdall, Claus Høgdall, Jan Blaakaer, Ellen L. Goode, Joellen M. Schildkraut, Douglas F. Easton, Susanne Krüger-Kjaer, Alice S. Whittemore, Simon A. Gayther, Paul D. P. Pharoah

**Affiliations:** 1 Gynaecological Cancer Research Laboratories, UCL EGA Institute for Women's Health, University College London, London, United Kingdom; 2 CR-UK Department of Oncology, University of Cambridge, Strangeways Research Laboratories, Cambridge, London, United Kingdom; 3 Department of Cancer Genetics, Roswell Park Cancer Institute, Buffalo, New York, United States of America; 4 Department of Health Research and Policy, Stanford University School of Medicine, Stanford, California, United States of America; 5 Institute of Cancer Epidemiology, Danish Cancer Society, Copenhagen, Denmark; 6 The Juliane Marie Centre, Rigshospitalet, University of Copenhagen, Copenhagen, Denmark; 7 Department of Gynaecology and Obstetrics, Aarhus University Hospital, Skejby, Aarhus, Denmark; 8 College of Medicine, Mayo Clinic, Rochester, Minnesota, United States of America; 9 Duke University Medical Center, Department of Community and Family Medicine, Durham, North Carolina, United States of America; 10 Genetic Epidemiology Unit, University of Cambridge, Strangeways Research Laboratory, Cambridge, United Kingdom; Ohio State University Medical Center, United States of America

## Abstract

**Background:**

Recent studies have identified several single nucleotide polymorphisms (SNPs) in the population that are associated with variations in the risks of many different diseases including cancers such as breast, prostate and colorectal. For ovarian cancer, the known highly penetrant susceptibility genes (*BRCA1* and *BRCA2*) are probably responsible for only 40% of the excess familial ovarian cancer risks, suggesting that other susceptibility genes of lower penetrance exist.

**Methods:**

We have taken a candidate approach to identifying moderate risk susceptibility alleles for ovarian cancer. To date, we have genotyped 340 SNPs from 94 candidate genes or regions, in up to 1,491 invasive epithelial ovarian cancer cases and 3,145 unaffected controls from three different population based studies from the UK, Denmark and USA.

**Results:**

After adjusting for population stratification by genomic control, 18 SNPs (5.3%) were significant at the 5% level, and 5 SNPs (1.5%) were significant at the 1% level. The most significant association was for the SNP rs2107425, located on chromosome 11p15.5, which has previously been identified as a susceptibility allele for breast cancer from a genome wide association study (P-trend = 0.0012). When SNPs/genes were stratified into 7 different pathways or groups of validation SNPs, the breast cancer associated SNPs were the only group of SNPs that were significantly associated with ovarian cancer risk (*P*-heterogeneity = 0.0003; *P*-trend = 0.0028; adjusted (for population stratification) *P*-trend = 0.006). We did not find statistically significant associations when the combined data for all SNPs were analysed using an admixture maximum likelihood (AML) experiment-wise test for association (*P*-heterogeneity = 0.051; *P*-trend = 0.068).

**Conclusion:**

These data suggest that a proportion of the SNPs we evaluated were associated with ovarian cancer risk, but that the effect sizes were too small to detect associations with individual SNPs.

## Introduction

One of the strongest risk factors for invasive epithelial ovarian cancer is a family history of the disease; a woman with a single first-degree relative diagnosed with ovarian cancer has a 2–3 fold increased risk [1]. Familial aggregation of cancer could be due to either a sharing of environmental or lifestyle risk factors within families, or through the inheritance of genetic risk factors. The higher rates of cancer in monozoygotic twins, compared with dizygotic twins or other siblings of individuals with cancer, suggests that genetic factors play a greater role [2].

Germline mutations in the high penetrance genes *BRCA1* and *BRCA2* are responsible for the vast majority of families containing multiple cases of ovarian cancer (>3 cases) and two or more cases of breast and ovarian cancer [3]–[5]. Other highly penetrant genes may exist, but these are likely to be rare and account for only a small fraction of the excess familial risk. The risk of ovarian cancer in first-degree relatives of ovarian cancer patients compared to the population risk is estimated to be about 2.4 [6]. Approximately 60% of this familial relative risk is not accounted for by the known high risk loci [7]. This remaining risk is likely to be caused by a combination of common low penetrance genes (the common variant: common disease hypothesis) and/or rare variants of moderate penetrance [8].

Genetic association studies, which compare the frequencies of common genetic variants between cases and unaffected controls, have become the preferred approach to look for low penetrance cancer susceptibility genes. Candidate gene studies have focused on common variants in genes that may play a role in cancer (e.g. cell cycle control; DNA damage repair and response; proliferation and apoptosis) with some success [9]–[14]. However, they have often been limited by small sample sizes, which reduce the power to detect associations at very stringent levels of significance. Consortium based studies have been used to increase the samples size and attempt to validate preliminary results [15]–[19]. More recently, genome-wide association studies, an approach that compares the frequency of hundreds of thousands of SNPs distributed evenly throughout the genome, have been successful in identifying low penetrance susceptibility variants for breast, colorectal and prostate cancers [20]–[25]. There are, as yet, no published genome-wide association studies for ovarian cancer. Therefore, we have continued to take a candidate gene approach to identify ovarian cancer susceptibility markers. We have mainly used an empirical approach, in which a minimal set of ”tagging” single nucleotide polymorphisms (SNPs) that efficiently capture all the common genetic variation in a gene, to find genetic susceptibility markers for ovarian cancer [11]–[14]. The candidate genes in this study were involved in several cancer related pathways; but we have also analysed candidate SNPs from the Ovarian Cancer Association Consortium (OCAC) validation studies and from two recently completed breast cancer genome wide association studies [20], [21].

The analysis of genetic association studies inevitably involves a large number of statistical tests, and there has been much debate about how to correct for multiple hypothesis testing. This has usually been considered a hypothesis-testing problem in which the aim is to control the overall “experiment-wise” type I error. The null hypothesis is that there is no association between any of the SNPs being tested with disease, and the aim is to test whether this global null hypothesis of no association can be rejected. A variety of methods have been proposed to test the global null hypothesis [26]–[30]. One of these, the admixture maximum likelihood (AML) test, simultaneously estimates the proportion of associated SNPs and their typical effect size. The power of the AML test has been found to be similar to or better than all other tests (rank truncated product, unrestricted maximum likelihood, restricted space maximum likelihood, most significant SNP, Global χ2, Best subset χ2) across a wide range of scenarios for the alternative hypothesis [31]. The simple Bonferroni correction performed best only when the number of associated SNPs was small (typically ≤3 or <5% of SNPs tested, whichever is smaller).

The AML method has been used to evaluate the overall evidence of association between 710 common variants in 117 candidate genes and breast cancer risk [32]. The results of this study showed that a proportion of SNPs in these candidate genes were associated with breast cancer risk, but that the effects of individual SNPs were likely to be small. In the current study, we use the AML method to evaluate data from 12 previous studies [9]–[19] for global evidence of associations between the risk of invasive epithelial ovarian cancer and 340 SNPs from 84 genes and 10 different chromosomal regions – SNPs in these regions are not within known genes or open reading frames. Three population-based studies comprising approximately 1,500 cases of invasive epithelial ovarian cancer and 3,100 unaffected controls were analysed.

## Materials and Methods

### Ethics Statement

Ethics committee approval was obtained for the collection and genetic analysis of all samples, and an informed written consent was obtained from all participants. Ethics approvals were granted by; the Danish Ethical Committees of Copenhagen and Frederiksberg (MALOVA), Anglia and Oxford Multi Centre Research Ethics Committee (SEARCH) and the Institutional Review Boards of Stanford University School of Medicine and Roswell Park Cancer Institute (GEOCS).

### Study individuals

Three population-based ovarian cancer case-control series were used in this research [9]: MALOVA (446 cases, 1,221 controls) from Denmark, SEARCH (719 cases, 855 controls) from the UK, and GEOCS (325 cases, 429 controls) from the USA. The case collection for MALOVA ran from 1994 to 1999 in Danish counties with a gynaecological hospital department. Cases were women diagnosed with invasive epithelial ovarian cancer aged 30–80 years. Controls were age-matched (within 3 years) and were randomly drawn from Danish born women in the study area by means of the computerized Danish Central Population Register. SEARCH began recruitment in 1998 and covers the regions served by the East Anglian and West Midlands cancer registries in the UK. Eligible women were those diagnosed since 1991 with invasive epithelial ovarian cancer under the age of 70 years. Controls, aged 45–74 years, from the same geographical region as the cases, were from the Norfolk constituent of the European Prospective Investigation of Cancer (EPIC) cohort. The Genetic Epidemiology Ovarian Cancer Study (GEOCS, formerly known as FROC) recruited participants from 6 counties in the San Francisco Bay area USA from 1997 to 2002. Patients, aged between 23–64 years, with invasive epithelial ovarian cancer were identified via rapid case ascertainment through the Greater Bay Area Cancer Registry operated by the Northern California Cancer Centre as part of the SEER Program. Control women were identified through random-digit dial and were frequency-matched to cases on race/ethnicity and five-year age group. Cases in MALOVA were collected prospectively, GEOCS retrospectively and SEARCH both prospectively and retrospectively. For all three studies, DNA samples were extracted from blood by Whatman International. Further details of these studies have been published previously [9]–[14].

### Gene and tag SNP selection

Candidate gene selection was primarily based on biological pathways that are predicted to be involved in ovarian carcinogenesis. The major pathways evaluated were DNA double strand break repair, cell cycle control and DNA mismatch repair (MMR). We also analysed several known or candidate tumour suppressor genes and oncogenes for ovarian cancer and a series of genes that we identified from the analysis of an *in vitro* functional model of ovarian cancer [33]. Finally, we analysed SNPs that have previously shown weak evidence of association with ovarian cancer risks and SNPs that are known to be associated with breast cancer risk. The pathways, genes and SNPs analysed are listed in Table S1. Further details on some of these genes and SNPs, and their association with ovarian cancer have been published [9]–[14].

For most genes, a tagging SNP approach was used to select known common variants. Haploview and Tagger were used for the selection of common variants from the reference CEPH genotypes. The approach involved the tagging of common SNPs with a tagging SNP (tSNP) with a minimum r^2^ of 0.8. If a SNP was poorly correlated with other SNPs, then 2- or 3-marker haplotypes were used, (an approach called “aggressive tagging”), if the haplotypes tagged the SNP(s) with a minimum r^2^ of 0.8. SNP tagging reduces the number of SNPs that require genotyping in association studies. The MMR gene study was completed before tagging SNP approaches were widely used due to the lack of available information from the International HapMap Project; and so we analysed SNPs of varying frequencies selected from public databases such as the dbSNP database (http://www.ncbi.nlm.nih.gov/SNP) and from the NIEHS Environmental Genome Project (EGP) (http://egp.gs.washington.edu/) [12].

The genotyping methods used in these studies have been described previously (9–14). All assays were carried out in 384-well plates and included 12 duplicate samples per plate (3%). Genotypes were excluded if duplicate concordance rates for a study were <98%. Plates also included non-template negative test controls. Finally genotypes were excluded from the analysis if call rates were <90% per plate. The average call rates for these SNP were 94% in cases and 96% in controls. A list of the SNPs genotyped in this study is provided in Table S1.

### Statistical methods

Associations between invasive epithelial ovarian cancer and each SNP were assessed using two tests; the one-degree of freedom Cochran–Armitage trend test and the general two-degrees of freedom χ^2^ test (heterogeneity test). Both tests were stratified by study to account for any differences within the sample sets. The overall evidence for an excess of associations between common variants and ovarian cancer risk was evaluated with the AML method, which is described in detail in Tyrer et al [31]. Briefly, the AML method formulates the alternative hypothesis in terms of the probability (α) that a given SNP is associated with disease and a measured effect size. When a SNP is associated with disease, the calculated χ^2^ statistic will be distributed, asymptotically, as a non-central χ^2^ distribution with the usual degrees of freedom and a non-centrality parameter η. The non-centrality parameter is a measure of the size of effect of the SNP, and is closely related to the contribution of the SNP to the genetic variance of the trait. The non-centrality parameter was assumed to be the same for all SNPs to make the model more parsimonious. This was an approximation but improves power if the non-centrality parameter is roughly the same for associated SNPs as fewer parameters have to be optimized. If η is assumed to be the same for each associated SNP, then both α and η can be estimated by maximum likelihood, and a test of the null hypothesis can then be obtained as a likelihood ratio test. In instances such as this, where some SNPs were correlated, pseudo-maximum likelihood estimates can still be produced by the same procedure, as if the SNPs were independent. Therefore the pseudo-maximum-likelihood method was applied to account for LD between SNPs. Simulation can subsequently be used to establish the statistical significance of the test. We applied the AML method using both the trend and heterogeneity tests. All analyses were adjusted for cryptic population stratification using the method described by Devlin *et al* [32], [33]. Genotyping data from 280 randomly selected, unlinked SNPs were used to adjust for population stratification using the genomic control method [33]. These genotyping data were from cases and controls derived from a breast cancer genome-wide association study [20]. The inflation test statistic we used was based on the inflation seen in genomic control samples for the breast cancer study and was chosen to be slightly higher than the estimated inflation as a “conservative” correction. We therefore adjusted the p-trend by 10% (1.1) and p-heterogeneity by 5% (1.05) as a means of conservatively allowing for any cryptic population stratification. Statistical significance is at the 5% level unless stated otherwise.

## Results

We have genotyped 340 SNPs in up to 1,491 invasive epithelial ovarian cancer cases and 3,145 unaffected controls from three different population based studies from the UK, Denmark and USA. SNPs were either tagging SNPs located in 84 candidate genes from pathways implicated in ovarian cancer development, or candidate SNPs located in 10 different regions on chromosomes 2, 3, 5, 8, 11, 12 and 17 that had been chosen for validation by the Ovarian Cancer Association Consortium (OCAC) or had been identified in a breast cancer genome-wide association study [9]–[21]. Genotype frequencies for these SNPs in cases and controls are given in Table S1.

**Figure 1:**
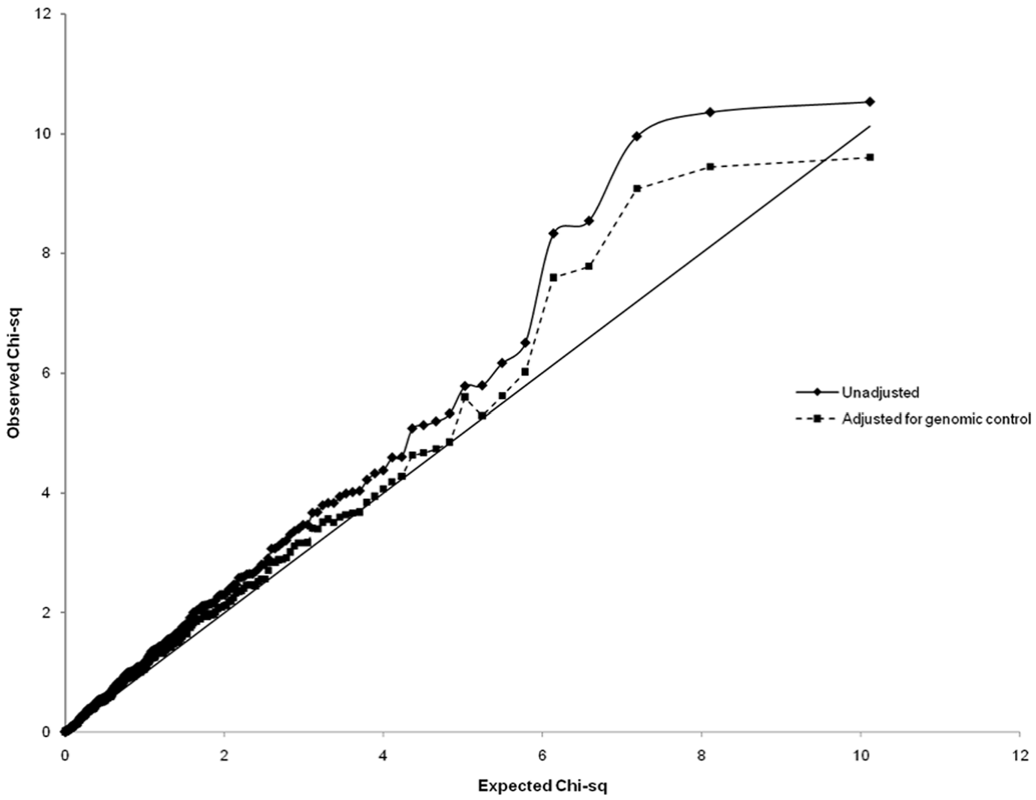
A quantile-quantile (Q-Q) plot of the univariate trend test results. The ordered test statistics are plotted against the expected statistics given the rank.

Using the trend test for association, 22 SNPs (6.5%) were significant at the 5% level, and 5 SNPs (1.5%) were significant at the 1% level. After adjusting for population stratification by genomic control, 18 SNPs (5.3%) were significant at the 5% level, and 5 SNPs (1.5%) were significant at the 1% level (Table 1). The test results for every SNP are given in Table S2. Figure 1 illustrates the results of the univariate trend test shown as a quantile-quantile (Q-Q) plot, in which the ordered test statistics are plotted against the expected statistics given the rank. The Q–Q plot follows the line of equivalence for the first 240 SNPs and then starts to deviate, as would be expected if a modest proportion of SNPs were associated with disease.

**Table 1:** All 22 SNPs with significant associations identified using the trend test for association.

Group	Gene location	rs number	MAF	Controls	Cases	HetOR‡(95% CI)	HomOR‡ (95% CI)	*P*-het*	Unadjusted *P*-trend	Adjusted *P*-trend§
BCAC	11p15.5	rs2107425	0.32	1460	2463	0.71 (0.62−0.82)	0.88 (0.70−1.10)	<0.0001	0.0012	0.0019
OCAC	*ESR*	rs9322336	0.23	1453	2464	0.81 (0.70−0.93)	0.73 (0.52−1.02)	0.005	0.0013	0.0021
BCAC	*LSP1*	rs3817198	0.3	1457	2435	1.16 (1.01−1.34)	1.40 (1.11−1.75)	0.006	0.0016	0.0026
Cell cycle	*CDKN1B*	rs2066827	0.26	1481	2484	0.88 (0.77−1.01)	0.68 (0.51−0.90)	0.011	0.0035	0.0053
Cell cycle	*CDK6*	rs8	0.21	1473	2481	1.17 (1.02−1.35)	1.44 (1.04−1.99)	0.015	0.0039	0.0059
Mismatch	*PMS2*	rs7797466	0.18	1305	1968	1.18 (1.01−1.38)	1.38 (0.96−2.00)	0.039	0.0108	0.0142
Cell cycle	*CCND1*	rs603965	0.44	1476	2464	1.06 (0.91−1.23)	1.28 (1.06−1.55)	0.027	0.013	0.0178
MMCT-18	*RUVBL1*	rs13063604	0.22	564	785	1.23 (0.98−1.56)	1.54 (1.00−2.39)	0.0556	0.016	0.0181
OCAC	*PGR*	rs1042838	0.14	1424	2408	1.25 (1.07−1.46)	1.09 (0.73−1.64)	0.019	0.0161	0.0215
Cell cycle	*CCND1*	rs7178	0.07	1480	2491	1.24 (1.04−1.49)	1.24 (0.50−3.04)	0.063	0.021	0.0278
OCAC	*IL18*	rs1834481	0.25	1449	2435	0.89 (0.77−1.02)	0.77 (0.59−1.01)	0.074	0.0227	0.0295
Cell cycle	*CCND1*	rs602652	0.46	1468	2493	1.13 (0.97−1.32)	1.24 (1.03−1.49)	0.074	0.0235	0.0307
OCAC	*11p15.55*	SNP1	0.2	1473	2402	0.84 (0.73−0.97)	0.86 (0.60−1.22)	0.0529	0.0243	0.0314
MMCT-18	*CASP5*	rs518604	0.44	1041	2029	1.11 (0.93−1.33)	1.27 (1.02−1.58)	0.0987	0.032	0.0387
Cell cycle	*CCND1*	rs3212879	0.49	1472	2491	0.85 (0.73−0.99)	0.82 (0.68−0.99)	0.063	0.0321	0.0409
DNA repair	*XRCC2*	rs3218536	0.08	1337	1787	0.88 (0.72−1.08)	0.23 (0.07−0.79)	0.014	0.0364	0.0439
Cell cycle	*CCND1*	rs3212891	0.46	1475	2476	0.86 (0.74−1.00)	0.83 (0.69−1.00)	0.082	0.0376	0.0472
Mismatch	*PMS1*	rs256563	0.12	1456	2446	2.50 (0.99−6.33)	2.15 (0.84−5.48)	0.0435	0.04	0.04
BCAC	8q24.21	rs10808556	0.4	1462	2453	1.15 (0.99−1.33)	1.20 (0.99−1.46)	0.1071	0.0446	0.0552
Cell cycle	*CDKN2A*	rs3731257	0.26	1480	2476	0.89 (0.78−1.03)	0.80 (0.60−1.07)	0.1345	0.0451	0.056
Cell cycle	*CCNE1*	rs3218036	0.31	1476	2481	1.07 (0.93−1.23)	1.27 (1.01−1.59)	0.1126	0.0458	0.0567
OCAC	*11p15.55*	SNP2	0.49	1459	2407	1.20 (1.02−1.40)	1.20 (1.00−1.44)	0.0611	0.0473	0.0581

‡ compared with common homozygous.

HetOR – heterozygous odds ratio, HomOR – homozygous odds ratio.

CI – confidence interval.

* P-heterogeneity.

§ Adjusted for population stratification.

The P-trend looks for a trend between the OR and the heterozygous (Het); and rare homozygous (Hom) when compared with the common homozygous; the P-heterogeneity (P-het) does not assume a correlation with increasing number of rare allele.

Of the 22 SNPs that were significant at the 5% level, three were SNPs that had been selected because of their association with breast cancer in genome wide association studies (of 16 in that group), eight were from the cell-cycle control pathway (of 101), two were from the DNA mismatch repair pathway (of 43), one was from the DNA double strand break repair pathway (of 28), two were from the MMCT-18 (functional candidate gene) group (of 63) and five were from the OCAC group of SNPs (of 55). However, no single SNP reached a level of significance to provide definitive evidence of association - the most significant association was for a breast cancer associated SNP, rs2107425, located on chromosome 11p15.5 (unadjusted *P*-trend = 0.0012). The SNP was still significantly associated with ovarian cancer risk after adjustment for population stratification (*P*-trend = 0.0019).

Table 2 shows the results of the AML experiment-wise tests summarised for the complete set of SNPs categorised according to functional group, biological pathway or genotyping group. The test for overall association was significant for the breast cancer associated group of SNPs identified by genome wide association studies (*P*-het. = 0.0003, *P*-trend = 0.0028; adjusted *P*-trend = 0.0059). No other group of SNPs was significant. When all the data were combined, the AML experiment-wise test for association was not significant for both the heterogeneity test (*P* = 0.051) and the trend test (*P* = 0.068). This suggests that, although not statistically significant, there is a trend towards a proportion of the SNPs evaluated being associated with disease and that the effect sizes were too small to detect for individual SNPs.

**Table 2:** AML experiment-wise test results for genotyping groups.

Pathway/Group	Genes/regions‡	No. SNPs	LR *P*-trend of most significant SNP*	AML *P*-het*	AML *P*-trend*	Reference with original single SNP analysis
BCAC†	5 (5§)	16	0.0012	0.0003	0.0028	*Song et al*. 2009 [19]
OCAC†	36 (6§)	55	0.0014	0.863	0.806	Ramus *et al*. 2008; [16]; Pearce *et al*. 2008 [15]; Palmeieri *et al*. 2008 [17]
MMCT-18	9	63	0.016	0.609	0.468	*Quaye et al*. *in preparation*
Cell cycle control	15	101	0.0035	0.274	0.225	DiCioccio *et al*. 2004 [9]; Song *et al*. 2006 [11]; Gayther *et al*. 2007 [14]
Mismatch repair	7	43	0.0106	0.706	0.702	Song *et al*. 2006 [12]
DNA repair	7	28	0.0374	0.366	0.444	Auranen *et al*. 2005 [10]; Song *et al*. 2007 [13]
Ovarian Cancer Oncogenes	5	34	0.0671	0.524	0.528	Quaye *et al*. 2009 [18]
**Total**	**84 (10)**	**340**		**0.051**	**0.068**	

* Based on GEOCS, MALOVA and SEARCH genotypes.

‡ SNPs in regions with no known genes or open reading frames are in parenthesis.

† candidate SNPs validated from the Breast Cancer Association Consortium (BCAC) and Ovarian Cancer Association Consortium (OCAC).

§ different SNPs from 8q24.21 were genotyped in both BCAC and OCAC sets; LR – logistic regression; AML – admixture maximum likelihood; het – heterogeneity.

## Discussion

There are many studies in the published literature describing a candidate SNP/gene approach to search for common, germline genetic variants associated with epithelial ovarian cancer risk. These studies provide some evidence of association with disease risk for some SNPs [9]–[19], [34], [35]. In the current study, we have used data from 12 of these studies from three population based ovarian cancer case-control series. In total, 340 SNPs in 94 genes or regions were analysed in approximately 1,500 cases and 3,100 controls. Based on results of univariate analyses, we found borderline evidence of association for several SNPs, but no susceptibility alleles significant at the *P*<0.00001 level, which has been suggested as the threshold for candidate gene studies [36]. In the current analysis, the most significant SNPs identified in this dataset were rs2107425 on chr 11p15.5 (*P = *0.0012) and rs3817198 in *LSP1* (*P = *0.0016), both of which have been identified as susceptibility alleles for breast cancer, and rs9322336, which is located in the oestrogen receptor (*ESR1*) gene (*P = *0.0013). All three SNPs remained significant after adjusting for population stratification.

Even though none of the associations we found were highly statistically significant, we cannot rule out that one or more of these SNPs, or alternative SNPs within the candidate genes we analysed were associated with ovarian cancer risk. The combined sample size from the three case-control studies did not have sufficient statistical power to detect associations with highly stringent levels of statistical significance. For individual variants, the statistical power of the study depends on the minor allele frequency, the risks conferred, and the genetic model. For this study, we had 97% power at the 5% significance level to detect a co-dominant allele with a minor allele frequency of 0.3 that confers an odds ratio of 1.2, and 96% power to detect a dominant allele with a minor allele frequency of 0.1 that confers an odds ratio of 1.3. For the top three SNPs, we used Pupasuite PupaSNP (http://pupasuite.bioinfo.cipf.es/) [37] to look for additional evidence that they may be involved in cancer aetiology, but found nothing striking. rs2107425 is located in a region of chromosome 11p15.5 which has no known genes or open reading frames; but it tags another SNP (rs2251375) with r^2^ = 1 that is in a region conserved in mice. rs3817198 is in the lymphocyte-specific protein 1 (*LSP1*) gene, also on chromosome 11p15.5, and also in a region conserved in mice. Loss of heterozygosity in this region has been found in ovarian, breast, lung, stomach and bladder cancers, and has been described as a tumour suppressor region in lung and breast cancers [38]–[44]. rs9322336 is in intron 2 of *ESR1*; no other common variants appear to tag this SNP. *ESR1* is a ligand activated transcription factor, which has been implicated in ovarian and breast cancer [45], [46].

Simple multiple testing correcting methods such as the Bonferroni or Sidak are too stringent for adjusting the results for the individual SNPs. Neither of these methods take into account the correlation that exists between SNPs that tag the genetic variation across gene regions. Therefore the results reported have not been adjusted for multiple testing. However, these SNPs may not be significant after adjusting for multiple testing. We have recently shown that methods that take into account the totality of the data have greater power to detect associations [33]. Therefore, we used the AML method to test the hypothesis that subsets of the SNPs we evaluated, rather than individual SNPs, were associated with ovarian cancer risk. We did not find evidence for an overall association between common genetic variation in the 94 candidate genes or regions and ovarian cancer risk. However we found evidence of an association for SNPs that had been identified from breast cancer association studies. This is intriguing given previous studies that have shown strong genetic links between breast and ovarian cancer for the highly the penetrant genes *BRCA1* and *BRCA2* [3] and recent association studies that suggest some common variants may be low-penetrance susceptibility alleles for multiple phenotypes (i.e. pleiotropism). For example SNPs in a region of chromosome 8q24 appear to be associated with breast, prostate and ovarian cancer risk [47].

In conclusion, the only significant global association identified was with the breast cancer associated SNPs. However, providing definitive evidence that any of these variants represents a true susceptibility allele is limited by the sample size and consequently the statistical power to identify risk alleles for which the effect size is likely to be small. It must also be considered that many associations are not validated in a second stage study. Genome wide association studies have successfully identified common risk alleles for some common cancers, including breast and prostate cancers [20], [48]. In both studies, the sample sizes were much larger than those used in the current study. These studies also showed that empirical genome-wide studies represent an efficient approach to identifying low-penetrance susceptibility alleles, but that the most significant alleles only confer small relative risks (<1.3). It will require larger numbers from more extensive multi-centre collaborations to find ovarian cancer susceptibility alleles that confer such modest risks. This has recently been achieved with the development of the ovarian cancer association consortium (OCAC), which comprises more than 20 ovarian cancer case-control studies throughout the world [49]; but as the experiences of the breast and prostate consortia show, common risk alleles for ovarian cancer are likely to exist and a combination of both candidate gene and genome wide association studies have the potential to identify them. However, it has so far proved difficult to identify highly significant SNPs for ovarian cancer using the candidate gene approach. GWAS studies of other cancers have shown that this is likely to prove a more successful approach to identify low-moderate risk alleles for ovarian cancer in the future.

## Supporting Information

Table S1 Genotype frequencies for all SNPs in cases and controls
(0.07 MB XLS)

Table S2 The logistic regression results for all SNPs.
(0.36 MB XLS)
